# Lateral Distribution of NBD-PC Fluorescent Lipid Analogs in Membranes Probed by Molecular Dynamics-Assisted Analysis of Förster Resonance Energy Transfer (FRET) and Fluorescence Quenching

**DOI:** 10.3390/ijms131114545

**Published:** 2012-11-08

**Authors:** Luís M. S. Loura

**Affiliations:** 1 Faculty of Pharmacy, University of Coimbra, Health Sciences Campus, Azinhaga de Santa Comba, 3000-548 Coimbra, Portugal; E-Mail: lloura@ff.uc.pt; Tel.: +351-239-488-485; Fax: +351-239-827-126; 2 Centre for Chemistry-Coimbra, Rua Larga, 3004-535 Coimbra, Portugal

**Keywords:** DPH, DPPC, fluorescence, FRET, lipid bilayer, membrane probe, molecular dynamics, NBD lipid

## Abstract

Förster resonance energy transfer (FRET) is a powerful tool used for many problems in membrane biophysics, including characterization of the lateral distribution of lipid components and other species of interest. However, quantitative analysis of FRET data with a topological model requires adequate choices for the values of several input parameters, some of which are difficult to obtain experimentally in an independent manner. For this purpose, atomistic molecular dynamics (MD) simulations can be potentially useful as they provide direct detailed information on transverse probe localization, relative probe orientation, and membrane surface area, all of which are required for analysis of FRET data. This is illustrated here for the FRET pairs involving 1,6-diphenylhexatriene (DPH) as donor and either 1-palmitoyl,2-(6-[*N*-(7-nitrobenz-2-oxa-1,3-diazol-4-yl)amino] hexanoyl)- *sn*-glycero-3-phosphocholine (C6-NBD-PC) or 1-palmitoyl,2-(12-[*N*-(7-nitrobenz-2-oxa-1,3-diazol-4-yl)amino]dodecanoyl)-*sn*-glycero-3-phosphocholine (C12-NBD-PC) as acceptors, in fluid vesicles of 1,2-dipalmitoyl-*sn*-3-glycerophosphocholine (DPPC, 50 °C). Incorporation of results from MD simulations improves the statistical quality of model fitting to the experimental FRET data. Furthermore, the decay of DPH in the presence of moderate amounts of C12-NBD-PC (>0.4 mol%) is consistent with non-random lateral distribution of the latter, at variance with C6-NBD-PC, for which aggregation is ruled out up to 2.5 mol% concentration. These conclusions are supported by analysis of NBD-PC fluorescence self-quenching. Implications regarding the relative utility of these probes in membrane studies are discussed.

## 1. Introduction

Because of its strong intermolecular distance dependence (and therefore sensitivity to spatial distribution), Förster resonance energy transfer (FRET) has multiple applications in membrane biophysics, such as membrane protein mapping, lateral heterogeneity (membrane domains), determination of the transverse location (depth) of fluorescent residues/labels inside the membrane, protein/lipid selectivity (preference of a specific lipid for the protein vicinity), and membrane protein oligomerization [1], both in spectroscopic studies and, more recently, under the microscope [2]. Even though FRET is still commonly used as a qualitative indicator of chromophore proximity without accounting for its actual kinetics, full quantitative exploitation of its potential involves modeling of the FRET observables. To this effect, the decay of donor fluorescence in the presence of an acceptor is especially useful. For FRET in bilayers, the actual form of the decay is complex, since it may involve multiple donor-acceptor interactions, with the probes separated by varying distances. Equations for the decay in several situations (donor and acceptor located at varying depth in the bilayer, with and without phase separation) have been described in the literature [3]. One of the simplest possible scenarios is the analysis of FRET data with the formalism for uniform planar distribution of fluorophores in a monophasic bilayer. In this case, the donor fluorescence decay (*i_DA_*(*t*)) is given by:

(1) $$i_{DA}(t)=exp\left(-\frac{t}{\tau_{0}}\right)exp\left\{-\pi R_{0}^{2}c\gamma \left[\frac{2}{3},{\left(\frac{R_{0}}{R_{e}}\right)}^{6}\left(\frac{t}{\tau_{0}}\right)\right]{\left(\frac{t}{\tau_{0}}\right)}^{1/3}\right\}.exp\left\{\pi R_{e}^{2}c\left(1-exp\;\left[-{\left(\frac{R_{0}}{R_{e}}\right)}^{6}\left(\frac{t}{\tau_{0}}\right)\right]\right)\right\}$$

In this equation, *R*_0_ is the critical distance for the FRET interaction (Förster radius), τ_0_ is the donor lifetime in absence of acceptor (and therefore exp(−*t*/τ_0_) is the donor decay in absence of acceptor), *c* is the number of acceptors per unit area, γ is the incomplete gamma function, and *R*_e_ is the distance of closest approach between donor and acceptor molecules. Although Equation 1 was originally derived for a plane of acceptors containing the donor (*cis* transfer), it is also valid if the donor molecule is separated from the acceptor plane by a distance *R*_e_, a situation common for membranes, as donors and acceptors are often located at different depths in the bilayer (*trans* transfer). Upon preparation of lipid vesicles, donor and acceptor molecules are frequently inserted in either of the bilayer leaflets, with equal probability. In this case, one must consider two planes of acceptors for a given donor, one corresponding to the acceptors lying in the same bilayer leaflet as the donor, and another for those located in the opposite leaflet. The decay law in this case is obtained by simply multiplying the intrinsic donor decay by the FRET terms corresponding to each plane of acceptors. Another common occurrence in membrane systems is a complex decay of donor even in the absence of acceptors, with a sum of two or three exponentials being required for a proper description. In this case, the above equation can be still used, provided that the exponential donor intrinsic decay term is replaced by this function, and τ_0_ is replaced by the average decay lifetime.

This was first applied to bilayer systems (unilamellar vesicles of egg yolk PC) in the pioneering study of Fung and Stryer [4]. These authors verified that even though there was excellent agreement between the experimental decays and the theoretical expectations for low acceptor concentrations, deviations become apparent for higher acceptor loads. This shows that, right from the very first FRET application to bilayer systems, several important features were revealed: first, FRET in fluid bilayers was overall well described by the analytical two-dimensional formalism; second, deviations could be interpreted in terms of non-homogeneous fluorophore distribution. From time-resolved data of FRET from *N*-(7-nitrobenz-2-oxa-1,3diazol-4-yl)-1,2-dipalmitoyl-*sn*-3-glycerophosphoethanolamine (NBD-DPPE) to *N*-(lissamine™-rhodamine B)-1,2-dipalmitoyl-*sn*-3-glycerophosphoethanolamine (Rh-DPPE) in 1-palmitoyl,2-oleoyl-*sn*-3-glycerophosphocholine [5], a linear variation of the recovered acceptor concentration parameter as a function of the acceptor:lipid ratio was verified, as expected, allowing the calculation of the area per lipid molecule. This dependence was also verified in FRET from octadecylrhodamine B (ORB) to 1,1′,3,3,3′,3′-hexamethylindotricarbocyanine [DiIC_1_(7)] in fluid 1,2-dipalmitoyl-*sn*-3-glycerophosphocholine (DPPC) large unilamellar vesicles (LUV) [6] and the NBD-DPPE/N-Rh-DPPE pair in the same system [7]. However, in both instances, it was observed that higher probe concentrations (~1 mol%) led to worse quality of fitting with the uniform probe distribution model, probably as a result of acceptor aggregation.

**Figure 1:**
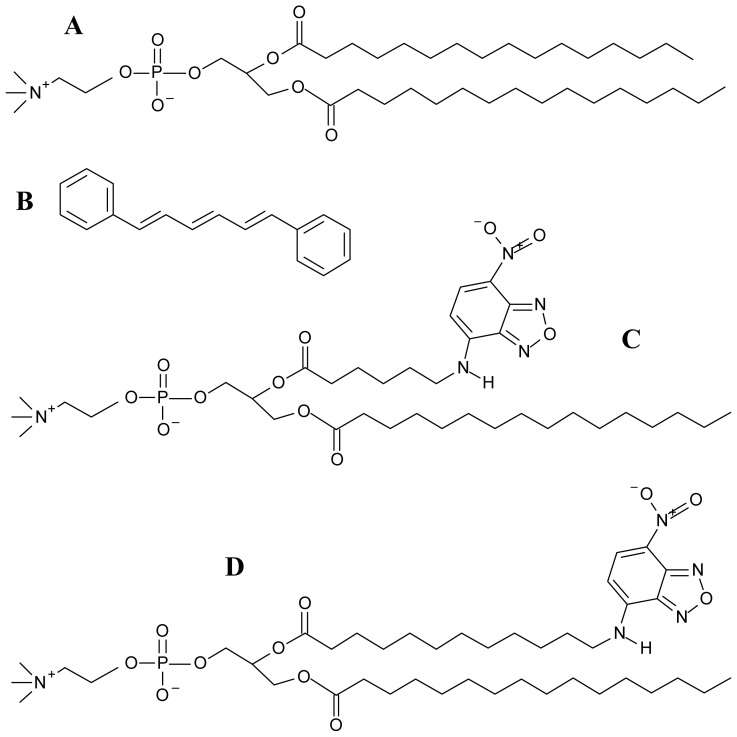
Structures of (**A**) 1,2-dipalmitoyl-*sn*-3-glycerophosphocholine (DPPC); (**B**) 1,6-diphenylhexatriene (DPH); (**C**) 1-palmitoyl,2-(6-[*N*-(7-nitrobenz-2-oxa-1,3-diazol- 4-yl)amino] hexanoyl)-*sn*-glycero-3-phosphocholine (C6-NBD-PC); (**D**) 1-palmitoyl,2-(12-[*N*- (7-nitrobenz-2-oxa-1,3-diazol-4-yl)amino]dodecanoyl)-*sn*-glycero-3-phosphocholine (C12- NBD-PC).

This work addresses FRET between 1,6-diphenylhexatriene (DPH) as donor, and either 1-palmitoyl,2-(6-[*N*-(7-nitrobenz-2-oxa-1,3-diazol-4-yl)amino] hexanoyl)-*sn*-glycero-3-phosphocholine (C6-NBD-PC) or 1-palmitoyl,2-(12-[*N*-(7-nitrobenz-2-oxa-1,3-diazol-4-yl)amino]dodecanoyl)-*sn*glycero-3-phosphocholine (C12-NBD-PC) as acceptors, in fluid bilayers of 1,2-dipalmitoyl-*sn*-3- glycerophosphocholine (DPPC, *T* = 50 °C; Structures in Figure 1). This setup was chosen because of both the wide use of these probes in fluorescence membrane studies [8,9] and the detailed characterization of their behavior in this lipid system using molecular dynamic (MD) simulations [10–14]. MD has been recently used as a tool to study the behavior (location, dynamics, effect on the host bilayer) of fluorescent membrane probes of various classes [15–17]. Given the large number of fitting parameters in the FRET analysis, it is convenient to fix as many degrees of freedom as possible, ideally using results from adequate biophysical techniques (such as MD), rather than merely following intuition. Therefore, this study is aimed at determining whether the probes are distributed uniformly in this membrane system at moderate concentrations, as well as illustrating the incorporation of structural data coming from atomistic MD systems to produce more accurate analysis of FRET time-resolved data.

## 2. Results and Discussion

### 2.1. FRET

The Förster radius, which is a measure of the intrinsic strength of interaction for a given FRET pair, depends on the relative orientation of donor and acceptor (through the so-called orientation factor, κ^2^; see [18] for an extensive discussion), the fluorescence quantum yield of donor in the absence of acceptor (Φ_0_), the refractive index of the medium (*n*), and the overlap between the donor normalized emission (*I*) and acceptor absorption (ɛ) spectra according to

(2) $$R_{0}=0.02108{\left[\kappa^{2}\Phi_{0}n^{-4}\int_{0}^{\infty }\lambda^{4}I(\lambda )ɛ(\lambda )d\lambda \right]}^{1/6}$$

**Figure 2:**
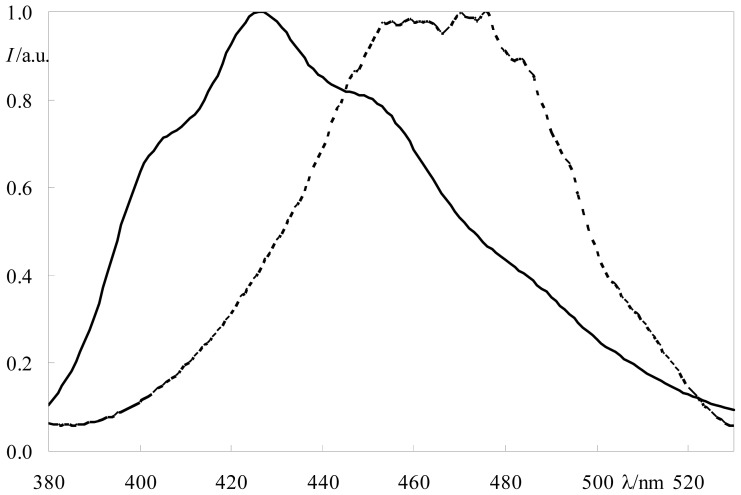
Corrected fluorescence emission spectrum of DPH (solid line) and excitation spectrum of C12-NBD-PC (dotted line) in DPPC large unilamellar vesicles (*T* = 50 °C).

**Figure 3:**
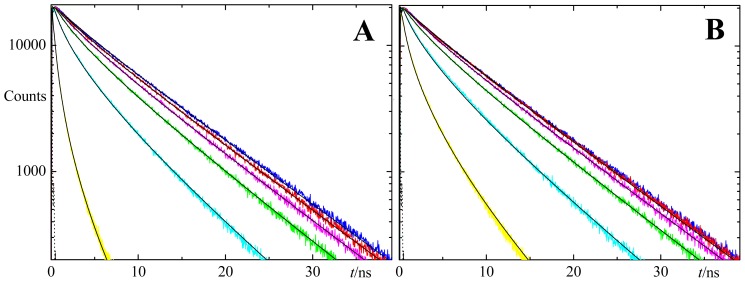
Fluorescence decays of DPH in presence of varying concentrations of (**A**) C6-NBD-PC (blue line: 0 mol%; red line: 0.040 mol%; magenta line: 0.10 mol%; green line: 0.24 mol%; cyan line: 0.62 mol%; yellow line: 2.5 mol%) and (**B**) C12-NBD-PC (blue line: 0 mol%; red line: 0.026 mol%; magenta line: 0.065 mol%; green line: 0.16 mol%; cyan line: 0.41 mol%; yellow line: 1.6 mol%). For each curve, the best global fits of model II (black solid lines; see text) are shown, as well as the instrument response function (black dotted line, visible for *t* < 1 ns).

The constant value in the equation above assumes that nm units are used for both λ and *R*_0_. Figure 2 shows in the same plot the emission spectrum of DPH and the excitation spectrum of NBD-PC, displaying a considerable extent of overlap, indicative of potential FRET interaction. Decays of DPH in DPPC fluid LUV (1:500 DPH:DPPC mole ratio, *T* = 50 °C), in the presence of varying amounts of acceptors, were recorded and are shown in Figures 3A (for the C6-NBD-PC acceptor) and 3B (for the C12-NBD-PC acceptor). As the acceptor mole fraction is increased, the decay becomes initially faster. As expected, at long times the decay curve becomes asymptotically parallel to that in the absence of acceptor, as the FRET rate (which scales as *t*^1/3^ for two-dimensional geometry) becomes negligible relative to the intrinsic decay rate (which scales as *t*).

As evidenced in Equation 1, quantitative analysis of the FRET decay curves requires precise knowledge of the interplanar donor-acceptor distance *R*_e_ and the Förster distance *R*_0_. The latter is used in the argument of the incomplete gamma function γ in Equation 1, and decay analysis allows in turn the retrieval of the parameter *nπR*_0_^2^, from which an output value of *R*_0_ (ideally consistent with the value used as input) is recovered, as will be illustrated below. From spectral data, and assuming the dynamic isotropic limit for the relative orientation distribution of both fluorophores (and hence <κ^2^> = 2/3), a value of *R*_0_ = 4.03 nm has been obtained for the DPH/NBC-PC pair [19]. On the other hand, due to its apolar structure, DPH could be anticipated to be deeply embedded inside the lipid bilayer [20,21]. A simple hypothesis, previously used in analysis of FRET data [19,22–24] would be to consider all DPH fluorophores as being equivalently located in the bilayer midplane. Regarding the transverse location of NBD-PC probes, diverging experimental values have been reported, Fluorescence quenching data analyzed with the parallax method indicate an external location of C12-NBD-PC, about 1.9–2.0 nm from the center of 1,2-dioleyol-*sn*-glycero-3-phosphocholine bilayers, and thus near the phosphate or even choline groups of the host lipids [25]. More recently, using NMR cross-relaxation rate measurements, Huster *et al.*[26] obtained a much shallower location, near the glycerol backbone/carbonyl region for both C12-NBD-PC and C6-NBD-PC. Given this uncertainty, an intermediate value of *R*_e_ = 1.7 nm could be considered as an *a priori* reasonable estimate for the distance between the donor and acceptor planes [19]. These *a priori* reasonable parameter choices are termed as analysis model I in the following discussion.

**Figure 4:**
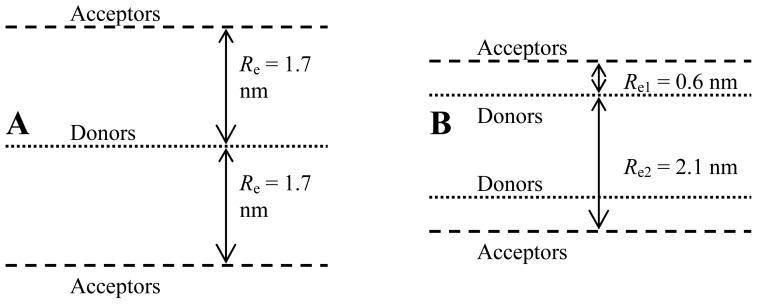
Schematic representation of the two topological models of probe distribution considered for analysis of Förster resonance energy transfer (FRET) decays. Model I (**A**) represents an intuitive approximate model (with DPH donors putatively located in the center of the bilayer, and NBD-PC acceptors in an interfacial location, consistent with some fluorescence quenching literature results) whereas Model II (**B**) incorporates information on the transverse distribution as revealed by atomistic molecular dynamics (MD) simulation (see text for details).

**Figure 5:**
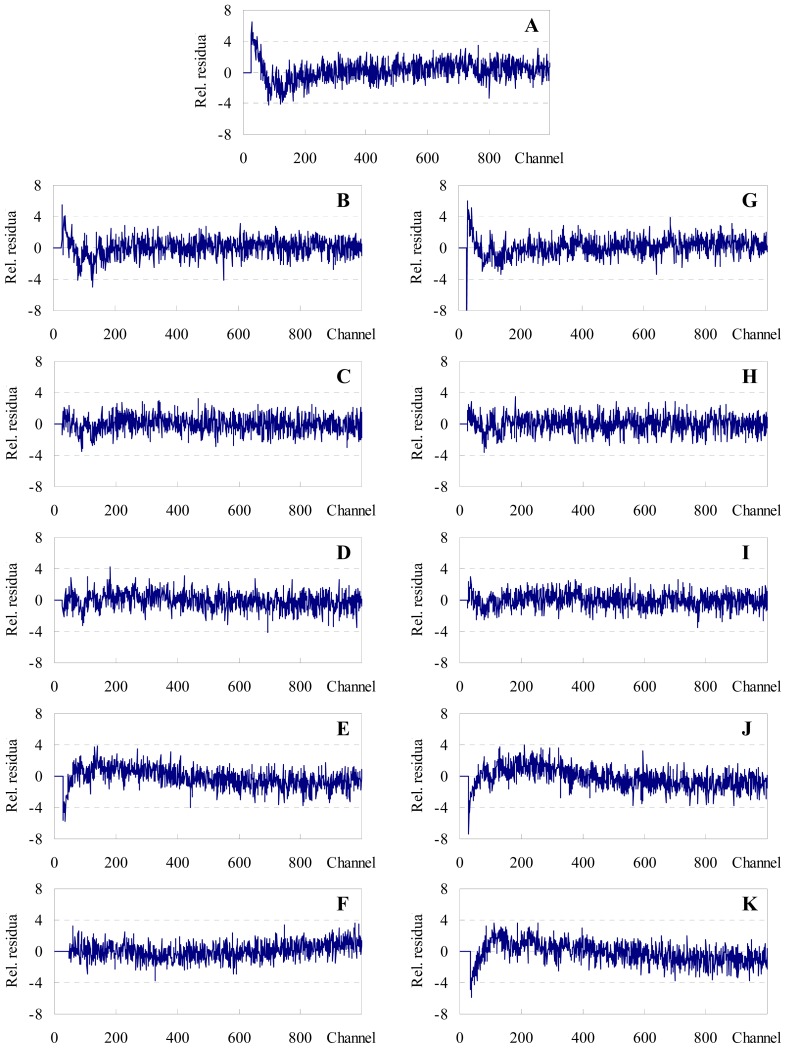
Residuals distributions of the fits shown in Figure 3. (**A**) no acceptor; (**B**–**F**): C6-NBD-PC acceptor, 0.040, 0.10, 0.24, 0.62 and 2.5 mol%, respectively; (**G**–**K**): C12-NBD-PC acceptor, 0.026, 0.065, 0.16, 0.41 and 1.6 mol%, respectively.

**Figure 6:**
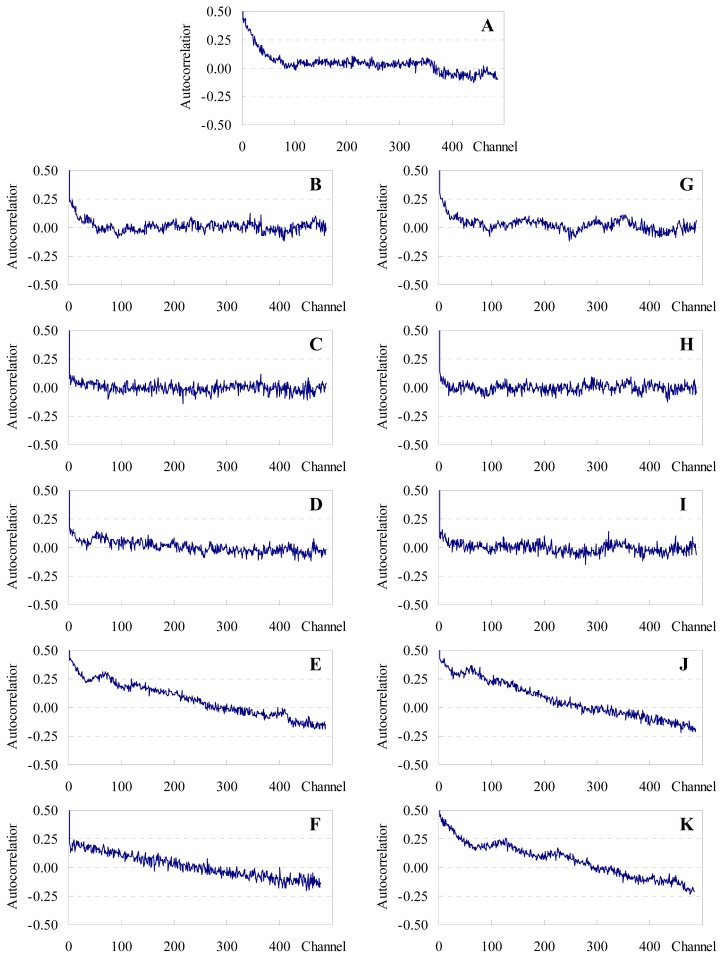
Autocorrelations of residuals of the fits shown in Figure 3. Panels as in Figure 5.

Alternatively, one may attempt to replace these educated guesses of *R*_0_ and especially *R*_e_ by making use of information recently obtained from MD simulations of both donor and acceptor. Repáková *et al.*[10] simulated DPH-labeled DPPC fluid bilayers (323 K). These authors found that the distribution of the angle between the polyene axis of DPH and the bilayer normal has a peak around *θ* ~25°, and the most probable location of the center of mass of DPH lies at ~0.75 nm from the bilayer midplane. On the other hand, we previously used MD simulations to study the behaviour of both C6-NBD-PC and C12-NBD-PC in the same lipid system [12]. We observed that the most probable orientation of the transition moment of the NBD chromophore (aligned with the short axis of the NBD group) occurred for *θ* ~125° (again, relative to the bilayer normal), whereas its most probable transverse location was at ~1.35 nm from the center of the bilayer. From this information, the method developed in the companion paper [27] leads to an average orientation factor of <κ^2^> = 0.66, virtually identical to the dynamic isotropic limit, and thus validating the use of *R*_0_ = 4.03 nm. On the other hand, consideration of the maximal transverse distribution locations for donor and acceptor leads to the assumption of two non-equivalent planes of acceptors for each donor, located at *R*_e1_ = 0.6 nm and *R*_e2_ = 2.1 nm relative to the donor plane (model II), as schematized in Figure 4. All eleven donor decays (one with no acceptor and five with varying concentration of each acceptor) were analyzed globally (with linkage of intrinsic donor decay parameters) in the framework of both models. The fits of model II are illustrated in Figure 3 together with the experimental decays (model I gives visually indistinguishable fit curves; results not shown), whereas distributions of the residuals and autocorrelation plots for this model are shown in Figures 5 and 6, respectively (model I results show minor differences, with slightly worse plots; not shown).

For each individual fit, the sole model parameter (apart from a multiplicative pre-exponential factor) is the acceptor concentration *C* = π*R*_0_^2^*c* (see Equation 1), which physically is the average number of acceptor molecules in a disk of radius equal to *R*_0_. The values of *C*, together with the χ^2^ values for each decay, as well as for the global analysis with both models, are given in Table 1.

From Table 1, it can be appreciated that whereas most individual decays are well described (χ^2^ < 1.5) by the global fit of model II, this is not verified for some of the samples with higher acceptor load, most importantly those with C12-NBD-PC as acceptor. This is also observed in the distribution of residuals (Figure 5) and (especially) autocorrelation plots (Figure 6), which show that distribution of residuals is mostly non-random for the samples most concentrated in the acceptor. Similar results have been observed by us in other systems, and are often interpreted as signs of acceptor aggregation [6,7,28,29]. Another sample for which the statistical description of the decays is surprisingly unsatisfactory is that without acceptor. Concerning this system, it should be mentioned that a single decay analysis gave acceptable statistics with a biexponential fit (τ_1_ = 2.09 ns (17%), τ_1_ = 8.55 ns (83%), χ^2^ = 1.18), as expected from literature reports [8], and pairwise linked analysis of this sample coupled with each of the acceptor-loaded samples also give satisfactory results (both looking at individual and global two-decay χ^2^ values), with the exceptions of the samples with 0.41 and 1.6 mol% C12-NBD-PC and that with 0.62 mol% C6-NBD-PC (not shown). Therefore, the worse statistical description (τ_1_ = 3.26 ns (16%), τ_1_ = 8.56 ns (84%)) of the decay without acceptors stems solely from the constraints imposed by linkage with decays which cannot be adequately fit by a model of FRET with uniform distribution. In any case, all individual χ^2^ values are < 2.0, and globally the condition χ^2^ < 1.5 is met.

The fits to model I give generally very similar results, with consistently slightly lower *C* and larger individual χ^2^. The exceptions are the two samples most concentrated in both acceptors, for which a significant improvement in χ^2^ (even if accompanied with only a slight change in *C*) is recorded when switching to model II. This results in the analysis with model I leading to ~10% larger global χ^2^ than model II (to a value > 1.5) and two individual χ^2^ > 2.0 values.

**Figure 7:**
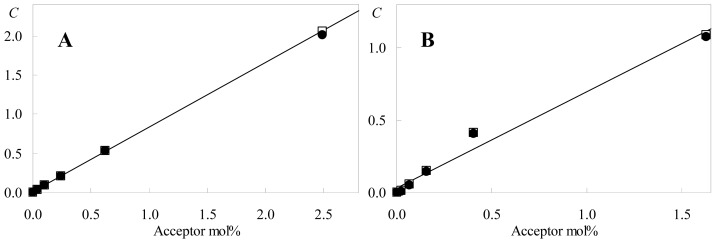
Variation of *C* = π*R*_0_^2^*c* values (recovered by fitting with models I (filled circles) and II (open squares)) with (**A**) C6-NBD-PC and (**B**) C12-NBD-PC acceptor concentration. Also shown are the best linear fits to the data obtained with model II.

A common verification of the underlying FRET theory in fits to time-resolved data is that of linearity between the recovered and analytical acceptor concentrations [4–7]. This is shown in Figure 7 for both donor-acceptor pairs with the two models.

As already evident from Table 1, for each donor/acceptor pair, the two models give largely identical results. Clearly, the fit-recovered and analytical acceptor concentrations have a very high degree of linear correlation in the C6-NBD-PC system (*r*^2^ = 1.000), and considerably less so in C12-NBD-PC (*r*^2^ = 0.982), for which negative curvature (another signpost of acceptor aggregation) is apparent. Moreover, the linear intercept is very close to zero (as physically expected) for DPH/C6-NBD-PC (0.008) but significantly larger for the other pair (0.033). From the slopes, *R*_0_ values for each pair may be retrieved, once the acceptor mole fraction abscissae *x* have been transformed into the corresponding numerical surface concentrations *c*. This can be easily done noting that *c* = *x*/*a*, where *a* is the average area/lipid molecule. The latter value of the parameter can be brought in either from the experimental literature, or, in the spirit of our approach, from MD simulations. In our aforementioned study of NBD-PC in fluid DPPC at 323 K [12], *a* = 0.667 nm^2^ was obtained. From this value, *R*_0_ = 4.18 nm is retrieved for DPH/C6-NBD-PC using model II (4.14 nm using model I), compared to 3.75 nm for DPH/C12-NBD-PC (3.74 nm using model I). Both recovered values are acceptably within ~10% of the input *R*_0_ values. The slightly higher, closer to the input *R*_0_ value recovered for C6-NBD-PC, accompanied by an excellent goodness of fit, is consistent with the absence of aggregation of acceptor probe in this system. Acceptor aggregation is a possible cause of the lower *R*_0_ value recovered for the DPH/C12-NBD-PC pair [6]. Overall, the recovered *R*_0_ values are close to those used as input, especially for C6-NBD-PC, and slight deviations may stem from uncertainty in the analytical concentration abscissa. Calculation of the latter depends on estimation of NBD-PC concentration from absorption in membranes and DPPC concentration from phosphorous analysis, both subject to experimental uncertainty. Note that apparent absorption coefficients of the NBD charge transfer band have been reported as highly variable and decreasing up to two-fold with decreasing polarity [30,31], which could, for example, explain the difference between the input and recovered *R*_0_ for the DPH/C6-NBD-PC pair.

### 2.2. Fluorescence Self-Quenching

**Figure 8:**
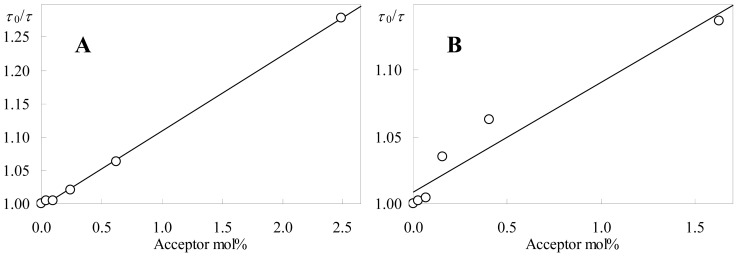
Time-resolved Stern-Volmer plots (Equation 3) of (**A**) C6- and (**B**) C12-NBD-PC. Linear fits to the data, with *k*_Q_ = 1.4 × 10^9^ (**A**) and 1.3 × 10^9^ mol^-1^dm^3^s^−1^ (**B**), are shown.

**Figure 9:**
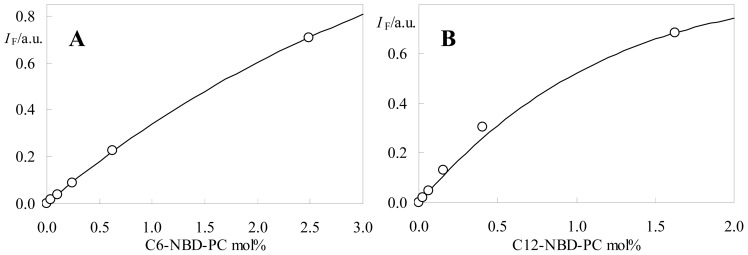
Concentration variation of the steady-state fluorescence intensity *I*_F_ of (**A**) C6-NBD-PC and (**B**) C12-NBD-PC. The lines are non-linear fits (of Equation 5, with *V* = 2.88 nm^3^ for C6-NBD-PC and 32.9 nm^3^ for C12-NBD-PC) to the data.

For a FRET-independent assessment of acceptor aggregation, we turn to the well known phenomenon of NBD fluorescence concentration self-quenching [7,32,33]. The variation of both time-resolved (Figure 8) and steady-state (Figure 9) NBD-PC fluorescence with probe concentration in the membrane was measured. The former was analyzed in terms of a simplified (taking into account average lifetimes) Stern-Volmer formalism:

(3) $$\langle \tau_{0}\rangle /\langle \tau \rangle =1+k_{\text{Q}}\langle \tau_{0}\rangle [\text{NBD}-\text{PC}]$$

where <τ> is the average lifetime, <τ_0_> is its value at infinite dilution, *k*_Q_ is the collisional quenching rate constant, and [NBD-PC] = *x*/*v*_DPPC_ is the fluorophore concentration (where in turn *v*_DPPC_ = 0.744 dm^3^mol^−1^ is the molar volume of DPPC at 50 °C [34]). The fluorescence lifetime of C12-NBD-PC in DPPC at 50 °C (τ= 4.0 ns for 2 mol% probe, and does not exceed 5.2 ns in DPPC in the 10–60 °C temperature range) has been reported [35], in agreement with our data point <τ> = 4.19 ns for 1.6 mol% probe. Surprisingly, given that the NBD fluorophore is expected to sense similar environments in the two probes, a clearly slower decay was observed for C6-NBD-PC (<τ_0_> = 6.00 ns) in comparison to C12-NBD-PC (<τ_0_> = 4.76 ns). Fluorescence lifetimes of C6-NBD-PC of ~7–8 ns have been measured in several different liquid disordered phases at 25 °C [36], and, specifically for fluid DPPC (48 °C), <τ> = 4.89 ns was measured in a tri-exponential fit for a 0.4 mol% probe (compared with our 5.64 ns value in a bi-exponential fit for 0.6 mol%) [37]. This short reported value is most probably affected by the vesicle labeling procedure by injection of the probe methanol solution (rather than co-solubilization, as done in our work). In fact, the major component (53%) is very short (0.81 ns) and, as discussed by the authors, due to C6-NBD-PC in a micellar state. Discounting this short component, one would obtain <τ> = 5.58 ns from the remaining two exponential terms, in very good agreement with our data.

As seen in Figure 8, whereas for C6-NBD-PC a good linear fit is obtained (with *k*_Q_ = 1.4 × 10^9^ mol^−1^dm^3^s^−1^; *r*^2^ = 0.999), this is not the case for C12-NBD-PC (with *k*_Q_ = 1.3 × 10^9^ mol^−1^dm^3^s^−1^; *r*^2^ = 0.939). These collisional quenching rate constant values may be related to the lateral diffusion coefficient (*D*) via the Smoluchowski equation for collision of molecules of a single species (and taking into account transient effects [38]):

(4) $$k_{\text{Q}}=4\pi N_{\text{A}}(2R)(2D)\left[1+2R/{\left(2D\langle \tau_{0}\rangle \right)}^{1/2}\right]$$

Here *N*_A_ is the Avogadro constant and *R* is the fluorophore collisional radius. Rather than try to calculate *D* from an assumed *R* value, we calculated what would be the required *R* to recover the MD-obtained NBD-PC *D* values ((11.7 ± 6.0) × 10^−8^ cm^2^s^−1^ for C6-NBD-PC, (8.3 ± 2.4) × 10^−8^ cm^2^s^−1^[12] for C12-NBD-PC), upon solving Equation 4. Virtually identical values of *R* = 0.77 nm and 0.78 nm are thus obtained for C6- and C12-NBD-PC, respectively. While these are not absurd values, they are somewhat higher than expected for the NBD collisional radius, probably indicating that completely efficient quenching is attained for slightly longer contact distances than predicted solely on the basis of the size of the NBD group estimated from atomic bond distances. Additionally, one should bear in mind that *D* values from MD are traditionally subject to considerable uncertainty, and hence these *R* values should be viewed cautiously.

In turn, the *k*_Q_ values obtained from time-resolved data may be used to investigate whether static quenching is operative. The equation for the variation of steady-state fluorescence intensity *I*_F_, allowing for both dynamic and static (active sphere model) self-quenching contributions, is given by (see, e.g., [39]):

(5) $$I_{\text{F}}=\frac{k[\text{NBD}-\text{PC}]}{1/\langle \tau_{0}\rangle +k_{\text{Q}}[\text{NBD}-\text{PC}]}\text{exp}(-{VN}_{\text{A}}[\text{NBD}-\text{PC}])$$

where *k* is a multiplying constant and *V* is the quenching active sphere volume.

From the fits with the data of Figure 9, the active sphere radii may be calculated. The value for C6-NBD-PC, 0.88 nm, is not dissimilar to the collisional radius calculated above, indicating that this probe does not aggregate to a significant extent in the studied concentration range. This result agrees with the reported linear variation observed for this probe’s steady state fluorescence in DPPC at 48 °C for concentration ≤1.5 mol% [37]. The opposite is verified for C12-NBD-PC. The recovered sphere radius (1.99 nm) is clearly indicative of a static quenching process related to probe aggregation, in agreement with the FRET data.

## 3. Experimental Section

### 3.1. Materials

The phospholipid DPPC and acceptor probes C6-NBD-PC and C12-NBD-PC were purchased from Avanti Polar Lipids (Birmingham, AL, USA). Donor probe DPH was obtained from Invitrogen (Carlsbad, CA, USA). 4-(2-hydroxyethyl)-1-piperazine sulfonic acid (HEPES), KOH and KCl (all from Merck, Darmstatdt, Germany) were used to prepare the buffer solution, 20 mM HEPES-KOH (pH 7.4) with KCl 100 mM. All organic solvents were of spectroscopic grade and came from Merck (Darmstatdt, Germany). Deionized water was used throughout. All above materials were used without further purification. The concentrations of stock solutions of probes were determined spectrophotometrically using the molar absorption coefficient values ɛ (DPH, 350 nm, in CH_3_OH) = 8.8 × 10^4^ M^−1^cm^−1^[40] and ɛ (NBD-PC, 465 nm, in C_2_H_5_OH) = 2.2 × 10^4^ M^−1^cm^−1^[40]. Final NBD-PC concentrations in vesicles were measured using ɛ (NBD-PC, 467 nm, in lipid) = 2.0 × 10^4^ M^−1^cm^−1^[41], whereas the final concentration of DPPC in vesicles was determined using phosphate analysis [42].

### 3.2. Preparation of Large Unilamellar Vesicles

Adequate amounts of stock solutions of DPPC (chloroform) and NBD probes (ethanol) were mixed in chloroform/methanol 2:1 (*v*/*v*), dried until complete evaporation (firstly by gently streaming nitrogen gas, followed by keeping in vacuum overnight), and suspended in buffer (HEPES 20 mM, pH 7.4) at 50 °C, to produce 0.5 mM lipid dispersions. Large unilamellar vesicles (LUV, ~100 nm diameter) were then prepared by extrusion of lipid dispersions through 100-nm pore diameter polycarbonate membranes as previously described [43]. DPH was added to pre-formed vesicles by injecting a very small volume (<0.1% of LUV suspension volume) of methanol stock solution, mixing thoroughly, and incubating at 50 °C in the dark for 2 h. The resulting lipid dispersions were stored at room temperature and used within 24 h of preparation. DPH:lipid ratio was kept as 1:500. Because of the large Stokes shift of DPH, homo-FRET among donor molecules (which would lead to faster FRET, as efficient homo-FRET would allow the possibility of quenching of a given donor fluorophore by a distant acceptor molecule following one or more migration steps) is inoperative. Should this not be the case, the donor:lipid ratio would have to be lowered even further to prevent this possibility, e.g., to 1:1000 for donor homo-FRET *R*_0_ of ~20–25 nm (such as NBD donors) or as low as ≈ 1:10000 for homo-FRET *R*_0_ of ~50 nm (such as BODIPY or rhodamine donors). On the other hand, at the very low fraction used, DPH does not aggregate and is essentially non-perturbing [11].

### 3.3. Instrumentation

Absorption spectroscopy was carried out with a Jasco V-560 spectrophotometer. When necessary, absorption spectra were corrected for turbidity using the method of Castanho *et al.*[44]. Steady-state fluorescence measurements were carried out with an SLM-Aminco 8100 Series 2 spectrofluorimeter in a right angle geometry with the cell holder thermostated at (50.0 ± 0.1) °C using a circulating water bath. The light source was a 450 W Xe arc lamp and the reference was a Rhodamine B quantum counter solution [39]. Correction of fluorescence spectra was performed using the correction software of the apparatus. 5 × 5 mm quartz cuvettes were used throughout. For the measurement of NBD-PC steady-state fluorescence intensity, excitation and emission wavelengths were 470 nm and 540 nm, respectively.

Fluorescence decay measurements were carried out with a time-correlated single-photon counting system, which is described elsewhere [45]. Excitation wavelength was 340 nm for both DPH (FRET) and NBD-PC (quenching). Emission wavelengths were 430 nm for DPH and 540 nm for NBD-PC. Timescales were chosen for each sample in order to observe the decay through 2–3 intensity decades. Instrumental response functions for deconvolution were generated from a scattering dispersion (silica, colloidal water suspension, Aldrich, Milwaukee, WI). Data analysis was carried out using in-house developed software implementing a non-linear least squares iterative convolution method based on the Marquardt algorithm.

### 3.4. MD Simulations

Because the MD simulations were published elsewhere, here we only describe in brief the protocol for our NBD-PC studies [12–14]. For the DPH simulations carried out by other authors, the reader is directed to the original references [10,11].

Runs of 100 ns (10 ns for equilibration, 90 ns for analysis) of 64 phospholipid molecules (one or four C6-NBD-PC or four C12-NBD-PC molecules, and 63 or 60 DPPC molecules, respectively) and 1947 SPC [46] water molecules were carried out. All calculations were performed with Gromacs 3.2 [47,48], under a constant number of particles, pressure (1 bar) and temperature (323 K), and with periodic boundary conditions. Pressure and temperature control were carried out using the weak-coupling Berendsen schemes [49], with coupling times of 1.0 ps and 0.1 ps, respectively. Semiisotropic pressure coupling was used. A united atom description of groups including hydrogen atoms was used and only one polar H atom per NBD-PC molecule was made explicit. All bonds were constrained to their equilibrium values, using the SETTLE algorithm [50] for water and the LINCS algorithm [51] for all other bonds, allowing the use of a time-step of 4 fs [52,53]. The adequacy of this time step and proper equilibration after the initial 10 ns were verified by inspection of plots of the time variation of temperature and energy. The long-range electrostatics Particle Mesh Ewald treatment [54] was applied. Van der Waals interactions were cut off at 0.9 nm. Parameters for bonded and nonbonded interactions of the DPPC molecule were taken from Berger *et al.*[55] For the C6-NBD-PC and C12-NBD-PC molecules, parameters were based on those for DPPC, except for the fluorophore atoms. For these, modified parameters were obtained as described in detail elsewhere [14].

## 4. Conclusions

This work addressed the distribution of the fluorescent lipid reporters C6-NBD-PC and C12-NBD-PC in fluid lipid bilayers (DPPC LUV, 50 °C), using state-of-the-art analysis of time resolved FRET data. The FRET donor used, DPH, is essentially non-perturbing at the very low fraction used (0.2 mol%) [11] and, therefore, eventual deviations from the theoretical decay law assuming uniform probe distribution could be ascribed to the acceptors. This probe and host lipid setting was also chosen given that detailed atomic-level topological and orientation information was available from MD simulation studies [10–14], which could be used as input in the FRET analysis. Several fluorescence approaches employing environment-sensitive probes (including those exhibiting excited-state intramolecular proton transfer (ESIPT)) may be useful in membrane characterization [56]. However, under normal conditions, these techniques do not provide a definite value for the distance of the fluorophore to the center of the bilayer, which is required as input for quantitative FRET analysis as used herein. In this regard, the most useful techniques probably are (i) differential fluorescence quenching using a family of quencher molecules with e.g., nitroxide spin-labeled analogs, having graded depths along the membrane (but note that there are in fact different formalisms for analysis of quenching data, with different assumptions, and sometimes yielding distinct results [57,58]); and (ii) molecular dynamics, which gives the fluorophore location distribution in a direct manner. Obviously, not many experimental researchers investigating FRET in membranes are inclined to carry out MD simulations of their systems in order to refine the FRET model parameters. But the comforting fact is that a number of fluorophores have been studied by atomistic MD simulations in the last 10 years or so and the list is continuously (albeit slowly) increasing [15,16]. MD is a most useful technique as it provides in a single “experiment” information on all structural aspects needed for consideration (probe transverse location and orientation, area/lipid), which otherwise would have to be obtained using several independent techniques, sometimes indirectly. However, it must be recognized that the quality of data from MD studies is highly dependent on efficient conformational sampling and correct force field and parameterization choices [15].

For analysis of the FRET data reported in this article, two models were considered: one based in the probe location and orientation conclusions of the MD studies, and one that took no account of the latter but relied on simple, “educated-guess” assumptions as used previously in the literature. The FRET data were complemented by a study of NBD-PC fluorescence self-quenching, for further elucidation of the aggregation states of the acceptor probes. Two main points should be emphasized.

Firstly, by comparing the results obtained with the two analysis models, it is clear that refinement of the topological/orientation models did not lead to considerable changes in the retrieved fit parameters. Notably, very similar acceptor concentrations were recovered for all samples. This is probably due to the fact that, despite the differences in donor-acceptor interplanar distance *h* between the two models, the latter were considerably smaller than the Förster distance *R*_0_ in both models. However, a modest but significant improvement in the statistical quality of fitting (as measured by the global χ^2^ values, which decreased by ~10%) was concomitant with the input parameter refinement. The better model description stemmed mostly from reductions in the individual χ^2^ values of the samples with higher acceptor concentrations. This highlights that even though the small refinements introduced in the FRET analysis inputs did not alter significantly the fitting parameters, the subtle changes in their best fit values produce a global χ^2^ improvement that could be the difference between rejection and acceptance of a given model. Therefore, while there is no basis to be overly suspicious of published results obtained with simple, “educated-guess” models, there is the danger that acceptable data may be rejected due to unsatisfactory statistical description caused by imperfect modeling, thus reinforcing the utility of introducing data from MD studies.

Secondly, taking this latter point as a cue, one may argue that the still high individual χ^2^ values obtained in the refined analysis for the most concentrated samples (especially for C12-NBD-PC) could result from a still non-optimal input model rather than from acceptor non-uniform distribution. For this reason, a study of the self-quenching of NBD-PC was carried out, as probe aggregation would most probably be reflected in a static self-quenching component. Dynamical self-quenching from Stern-Volmer analysis of NBD-PC lifetime data was consistent with lateral diffusion coefficients obtained from MD. On the other hand, static self-quenching was apparent for C12-NBD-PC but not for C6-NBD-PC. The overall conclusion is that while C6-NBD-PC distributes uniformly in fluid DPPC bilayers up to 2.5 mol%, C12-NBD-PC seems to aggregate for concentrations as low as 0.4 mol%. Unusual differences in fluorescence lifetimes (given the identical fluorophores, and the expected similar transverse location) were recorded for the two probes, even in dilute solution, with C12-NBD-PC presenting longer <τ> values. Together, the data indicate that C6-NBD-PC is a better-behaved membrane probe regarding uniformity of lateral distribution in fluid disordered bilayers. While our MD study gives no clear indication on the origin of this effect, it is possible that it may be related to conformational differences in the labeled *sn*-2 chains of the probes. For example, the NBD amine N atoms of both probes establish H bonds with phospholipid carbonyl and ester O atoms; however, whereas for C12-NBD-PC all H-bonds to lipid atoms are intermolecular, for C6-NBD-PC most of them are intramolecular. These differences in H-bonding pattern are reflected in distinct constraints on the conformation of the NBD fluorophore of the two derivatives. In particular, the labeled *sn*-2 chain of C6-NBD-PC does not extend nearly so much across the bilayer as that of C12-NBD-PC [12,13], probably remaining more shielded from interactions with other NBD fluorophores. In any case, this study indicates that C6-NBD-PC is better suited than C12-NBD-PC as a membrane probe for applications where aggregation should be avoided.

## Figures and Tables

**Table 1:** Fitting parameters for global analysis of Förster resonance energy transfer (FRET) decay data.

		Model I		Model II	
			
	mol% acceptor	*C*	χ^2^	*C*	χ^2^
	
No acceptor	0	0 (fixed)	1.97	0 (fixed)	1.92
C6-NBD-PC acceptor	0.040	0.032	1.45	0.034	1.43
	0.099	0.091	1.18	0.093	1.17
	0.24	0.207	1.23	0.210	1.21
	0.62	0.525	1.93	0.530	1.75
	2.5	2.011	1.89	2.054	1.19

C12-NBD-PC acceptor	0.026	0.0093	1.67	0.011	1.65
	0.065	0.053	1.24	0.055	1.23
	0.16	0.146	1.09	0.149	1.08
	0.41	0.407	2.06	0.411	1.95
	1.6	1.076	2.47	1.085	1.94

Global χ^2^		1.64		1.49	
